# A new education agenda based on The International Science and Evidence Based Education Assessment

**DOI:** 10.1038/s41539-024-00288-w

**Published:** 2025-05-10

**Authors:** Nandini Chatterjee Singh, Tal Gilead, Anya Chakraborty, Jo Van Herwegen, Nienke van Atteveldt, Gregoire Borst, Stephanie Bugden, Kaja Jasinska, Jonathan Kay, Kenneth Pugh, Anantha Duraiappah

**Affiliations:** 1 UNESCO Mahatma Gandhi Institute of Education for Peace and Sustainable Development (MGIEP), New, Delhi, India; 2 Seymour Fox School of Education, Hebrew University of Jerusalem, Jerusalem, Israel; 3 University College London, IOE - Psychology & Human Development, London, UK; 4 Department of Clinical, Neuro- and Developmental Psychology & Institute Learn!, Vrije Universiteit Amsterdam, Amsterdam, The Netherlands; 5 Laboratoire de Psychologie du Développement et de l’éducation de l’enfant (LaPsyDÉ -CNRS), Université de Paris, Paris, France; 6 Department of Psychology, The University of Winnipeg, Manitoba, Canada; 7 Applied Psychology and Human Development, University of Toronto, Toronto, Canada; 8 Education Endowment Foundation, London, UK; 9 Haskins Laboratories, New Haven, CT USA

**Keywords:** Education, Interdisciplinary studies

## Abstract

The International Science and Evidence Based Education Assessment examined whether current education systems develop each person’s full potential (aligned with the UN Declaration of Human Rights) and contribute to Sustainable Development Goal 4. Embracing a multidisciplinary approach, nearly 300 scientists from 45 countries conducted the assessment, calling for a shift in education’s focus from economic growth to fostering human flourishing. Key findings included (a) the need for an integrative approach to learning, (b) moving beyond meritocracy and exploring potentiality as a better measure of student learning potential, and (c) using technology judiciously for scalable, equitable, and personalised learning. This paper seeks to highlight themes that were foundational to the assessment but not fully discussed within it. It advocates a global, transdisciplinary research agenda to close evidence gaps and inform policy to consider the complexity of the educational system and the need to think beyond existing conventions.

## Introduction

**Fig. 1:**
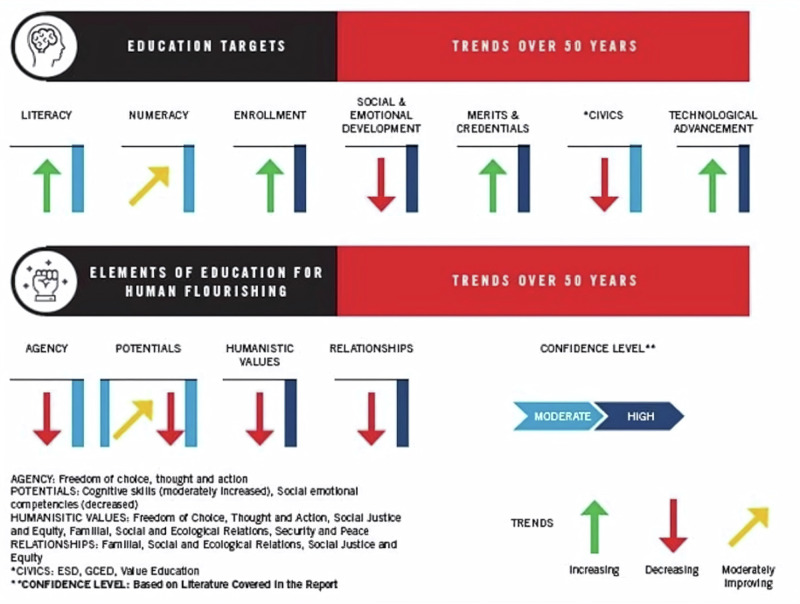
Trends in education targets and elements of education for human flourishing^1^. There has been a trend in the positive direction in education targets of literacy, numeracy, enrolment, merit, credentials, and technological advances (Fig. 1) while a trend in the negative direction in agency, humanistic values, relationships, and civic engagement^1^.

Current education systems embrace economic growth and financial prosperity as the primary paths to well-being and societal advancement^1–5^. Consequently, while the last few decades have shown an upward progression in literacy, numeracy, enrolment, merit, credentials, and technological advances (Fig. 1), it was mirrored by a downward trend in agency, humanistic values, relationships, and civic engagement^1^. Moreover, the priority given to developing individual skills and knowledge for economic productivity while neglecting holistic development^6^ seems also to have reinforced existing power structures and social disparities, favouring those with access to better-quality education or higher socioeconomic status^7^, and thereby ultimately contributing to increased inequality^8^.

Perhaps nothing demonstrates the dualism of education for economic development more clearly than the problem of inequality. While education has contributed to economic prosperity on a societal scale, the accompanying promises of equality have not been fulfilled. In human capital theory, which underpins the view that education is a tool for economic growth, meritocracy is identified as the mechanism through which education should promote social mobility^9^. This theory asserts that greater equality will result from an unbiased assessment of performance that genuinely reflects merit. The basic assumption is that if education is provided based on merit where meritocracy comprises academic skills delivered primarily through teachers, it will facilitate individual mobility within the social structure.

However, it has been demonstrated that an emphasis on individual mobility through meritocracy often perpetuates existing inequalities by overlooking structural barriers to success, such as disparities in access to quality education, healthcare, economic resources, and social support^10–12^. Recent figures on learning poverty^13^ paint a bleak picture, illustrating how education concentrated on achieving economic aims is contributing to the formation of a new aristocratic class, thereby exacerbating income inequality and fuelling social unrest. Furthermore, entrenched inequalities and social unrest exacerbate poverty, which is not merely a lack of income or material resources but is also linked to low educational attainment, poor health outcomes, social exclusion, and limited access to essential services like education, healthcare, housing, and sanitation^14–18^. As a result, despite increased school enrolments, there has not been a corresponding rise in equitable, high-quality learning opportunities for all. Instead, an education system driven by a meritocratic ideology has inadvertently deepened the issues of poverty and inequality rather than alleviating them.

Two significant lessons emerge from the cases discussed above. The first and broader lesson is that we must take into consideration the potential unintended consequences of education policy. We must acknowledge that education functions within a complex societal system that often behaves unpredictably due to its multiple disordered elements. Furthermore, many of the issues that education addresses can be categorized as “wicked problems”^19^. In the literature, this term refers to problems that are difficult to define, involve a high degree of uncertainty and inconsistency, and whose solutions often result in unexpected consequences. Climate change, inequality, and polarization are examples of such problems that education is expected to mitigate. Yet, these issues can actually be exacerbated by educational interventions due to their complex and unpredictable nature. The second, more focused lesson is that education’s pathway to well-being and societal advancement cannot solely rely on economic progress. To achieve these goals, education must adopt a broader perspective that considers a variety of contributing factors beyond the economic domain.

Taking the two above insights as its point of departure, the International Science and Evidence Based Education Assessment (ISEE Assessment)^20^, as laid out in the position paper^21^ sought to answer two main questions. First, are our education systems serving the “right” purpose? Second, can current education systems be reformed to address wicked problems?

In line with the Intergovernmental Panel on Climate Change (IPCC), the ISEE Assessment harnessed transdisciplinary expertise in learning and educational systems to assess education’s impact on developing human potential, fostering individual flourishing according to the Universal Declaration of Human Rights, and promoting peaceful, sustainable societies as outlined in SDG 4.7. This endeavour involved uniting scientists and experts from various disciplines and countries to compile a comprehensive assessment report on the scientific, technical, and socioeconomic knowledge of learning and education, identifying information gaps and future research priorities in education.

**Fig. 2:**
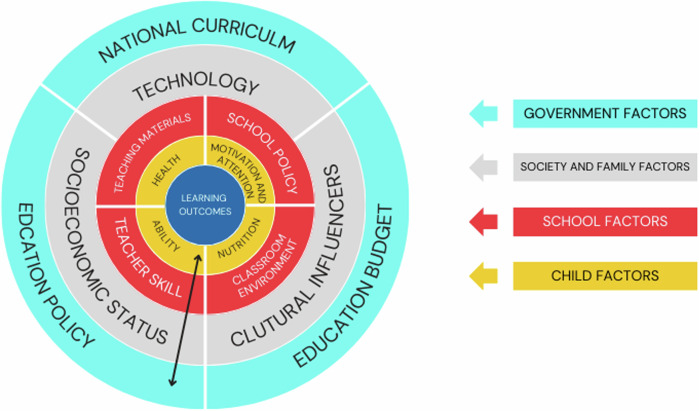
A multi-level model of education, inspired by Bronfenbrenner’s ecological systems model that links learning outcomes for a child with micro, meso and macro levels of the education system. The micro comprises child and school factors, society and family are meso factors, and the macro comprises government factors.

The report also sought to span the micro, meso, and macro levels of education to investigate the linkages of education to potentially arrive at recommendations that could inform education policy and governance (Fig. 2).

As a continuation of the position paper that laid out the purpose and conceptual framework of the ISEE Assessment^21^, the current paper describes key results of this report and lays out policy-relevant information and recommendations for a new social contract for education. It brings to the fore some central aspects of the ISEE Assessment that are particularly meaningful. However, it is important to clarify that this paper does not encompass all the recommendations from the original assessment, as these are detailed in the report itself^20^ and subsequent policy briefs^21^. Instead, the paper aims to offer a post-reflection on key principles that were not explicitly addressed within the assessment but have emerged from it and provide valuable insights for future educational policy design and formulation.

The result section defines key terms that emerged from and guided the assessment along with key findings and recommendations. A brief description of the conceptual framework and the process adopted for the ISEE Assessment is provided in the methodology^20^^,^ section. The discussion section presents steps for policy action and further research.

## Results

**Fig. 3:**
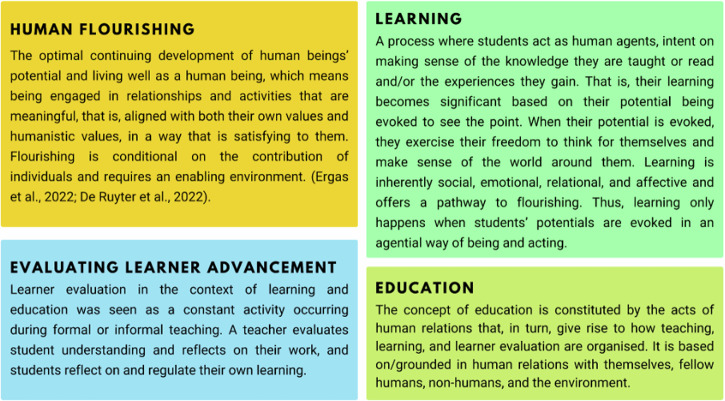
Working definition of human flourishing, learning, evaluation and education. The transdisciplinary research review and consensual discussions among the working groups led to fundamental descriptors of human flourishing, learning, learner advancement and education, against which the assessment was carried out.

Based on the definitions described in Fig. 3, the assessment arrived at three prominent findings, each of which will be discussed in a separate section.

1. An integrative learner-centric approach to teaching and learning
2. Potentiality (in place of meritocracy) in education systems
3. The evolving role of technology in education

The following section delves into the concept of an “integrative learner-centric approach.” It underlines the importance of adopting a holistic perspective over one based on compartmentalization. This section stresses the significance of interconnecting, merging, and viewing education as an integrated dynamic system, a key principle in the assessment.

The subsequent section recommends transitioning from a reliance on meritocracy to focusing on potentialities for advancing educational equality. This section emphasizes the significance of change, trajectories, transformation, and improvements. It underscores another foundational principle: education should concentrate on dynamic processes with open-ended outcomes rather than embrace a static worldview. The last section of the results presents the examination of how teachers can utilize technology, emphasizing the complexities, and the crucial role of context. This entails exploring the reciprocal relationships of education with the environment, characterized by mutual influences between these elements. The section underscores the need for education not only to adapt but also to influence and shape its surroundings proactively.

### An integrative learner-centric approach

The ISEE Assessment’s findings highlight the need to foster 21st-century skills for human flourishing. This extends beyond traditional academic skills of literacy and numeracy to explicitly include cognitive and social-emotional competencies^22^ (https://www.oecd.org/site/educeri21st/40756908.pdf). The evidence to support such an approach is transdisciplinary and broadens the understanding of what constitutes scientific approaches in education. Such an integrative approach to learning and teaching promotes skills and behaviours that cultivate individual and collective flourishing and foster a peaceful and sustainable mindset towards oneself, others, and the environment.

Learning occurs through social interactions, reorganisation of existing knowledge namely self-reflection or meta-cognitive skills as well as through experiential learning. Thus curricula and pedagogies should build and nurture social-emotional, metacognitive, and cognitive domains concurrently. Moreover, these do not develop or operate in isolation but dynamically interact with the environment to influence decision-making and behaviour^23–26^. In light of these findings, ISEEA proposes an integrative learner-centric approach that should be integrated to form the CASE^20,25^ (cognitive, academic, social, and emotional) approach across all levels of education—learning, teaching, and assessment.

While building social and emotional competencies is becoming more prevalent, it remains peripheral^27^ in its approach and acceptance and competencies such as teamwork, commitment, empathy and socialisation function in education are not emphasized. As outlined above, such lack of emphasis is in direct contrast with the scientific evidence demonstrating no clear demarcation of cognitive, social, and emotional processing in the human brain and how these domains influence the foundational skills of literacy and numeracy^2^.

Operationalizing the learner-centric approach requires an overhaul of curricula, shifting the emphasis away from learner assessments, and teacher monitoring towards localized curricula that tackle the existential questions students encounter in their daily lives^28,29^. Throughout the ISEE Assessment^2^, there are recommendations on how to actualize a learner-centric education system. These include:

- individualized learning where learning experiences are tailored to each student’s needs, interests, and abilities; the assessment recognizes that personalized learning is still in its early stages and achieving it as a goal necessitates the use of inventive and imaginative approaches for implementation.
- continuous and immediate feedback and assessment, such as formative and dynamic assessment for learning that uses self- and peer-assessments to facilitate learning;
- learning environments that foster critical thinking, problem-solving, and collaboration where diverse students can work together and learn from each other;
- teachers moving from knowledge providers to active participants to guide and support students’ learning journey and agency.

In summary, an ***integrative learner-centric*** or **CASE** approach strengthens the interconnectedness of cognition, academic and the social-emotional domains, which is essential for learning that contributes towards human flourishing^20,25^. The learner-centric approach is relevant to education for human flourishing as it empowers students to become independent lifelong learners who can adapt to new challenges and continue learning beyond formal educational settings. It acknowledges that students are active participants in their education and that their interests and motivations are critical drivers of learning.

### Beyond meritocracy

The ISSEA finds that the appeal to meritocracy as a great equalizer might be a barrier to education for human flourishing. Meritocracy involves allocating educational resources and opportunities based on merit, determined through performance assessments such as national standardized tests^7^. It ensures that the highest-performing students should receive access to higher education based on their achievements, irrespective of their social, cultural, ethnic, or economic background.

A detailed analysis reveals a significant fallacy in implementing meritocracy in education. Students from minority groups, such as low-income families or ethnic and religious minorities, often face significant disadvantages^30^, making allocating resources based on merit challenging for multiple reasons. One example is the failure to question the neutrality of assessments that may favour certain social groups.

**Fig. 4:**
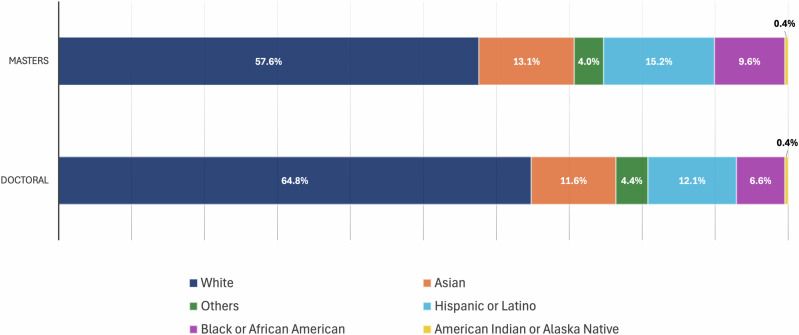
An example of meritocracy. Source - National Center for Science and Engineering Statistics, Survey of Graduate Students and Postdoctorates in Science and Engineering, 2021. The figure shows the underrepresentation of Hispanic, Black, and Native American students in science and engineering graduate programmes at master’s and doctoral level in the United States (https://ncses.nsf.gov/pubs/nsf23315/report/graduate-enrolment-in-science-and-engineering). Hispanic or Latino may be any race; race categories exclude Hispanic origin. Other includes Native Hawaiian and Other Pacific Islander and more than one race. In addition, there are disparities in educational attainment and pay. Hispanic adults have a 33% high school non-completion rate; 11% of Native American adults are unemployed; and Asian adults with bachelor’s degrees earned the most ($69,100), surpassing their white, black, and Hispanic peers^33^.

For instance, learners from different social groups have unequal access to material, social, and cultural resources. At school, learners from the dominant group are more familiar with curricula, learning strategies, language and interaction patterns, and assessments that set them up for success. At the group level, success is likely driven by these early advantages. Thus, the argument that meritocracy, as an ideal, does not exhibit structural bias is rejected by many scholars^31,32^ (Fig. 4 as an example from the United States^33^).

Instead, it can be argued that meritocracy as the great social equalizer has had the opposite effect. It has created a “new form of educational, social, and economic exclusion in the guise of credentialism and exacerbating inequitable flourishing outcomes” or what is known as “hereditary meritocracy”^2^ (p. 29). The failure of the merit-based model highlights the need to shift the focus from the “individual-deficit” model to a model that highlights the structural inequalities that meritocracy propagates.

Furthermore, educational policies have exacerbated inequality. Interventions often prioritize individual skills and neglect systemic barriers. Learners from diverse backgrounds need similar skills, but these manifest differently.

Advances in the science of learning have revealed that human learning and behaviour are functions of biology, opportunity, and environment^34^. Consequently, human beings show variation in learning potential^1^ and also exhibit individual differences in learning^7,34^. Based on this evidence, the ISEE assessment proposes potentiality rather than meritocracy as a measure of assessment for human flourishing. Potentiality is conceptualised as a personalised path that is dynamic and evolves with learning, varies across individuals and is shaped by educational environments. Some key principles that support such an approach are (a) While meritocracy traditionally focuses on academic achievement, a potentialities-based approach considers a broader range of factors that contribute to human flourishing; (b) The potentialities-based approach is dynamic and can be personalised. Thus, rather than a static, one-size-fits-all assessment system of meritocracy, which assumes equal opportunity, identical biology and similar environments, potentiality views education as a dynamic process that embraces growth.

The assessment approach of potentiality does not measure the absolute potential of each individual. Such a measurement is neither achievable nor suggested by the assessment Instead, it proposes to operationalise the concept of potentiality as a measure of an individual’s learning rate, which is assessed through dynamic, formative assessments that reflect personal learning trajectories. This model focuses on individual progress over time rather than comparison with others. It shifts the emphasis from competing for resources based on static criteria to nurturing each learner’s development potential.

Thus, transitioning from a meritocracy-based system to one focused on potentiality represents a shift from a static one to one that is dynamic, continuous and focuses on individual growth trajectories where growth is measured as a change from a former state to a new state, for an individual and not across individuals. Such an approach recognises that education requires frameworks that capture its evolving nature. It also calls for recognition that education is complex and needs to be adaptive so that it can continuously respond to the diverse and shifting needs of learners over time.

### Teachers and technology

The ISEE Assessment highlights technology’s dual role in education based on design, context, and implementation. Evidence shows technology’s potential in facilitating human flourishing^26,35,36^. However, when technology is designed and implemented without caution, it can impede desired learning outcomes^37,38^.

Although access to technology in schools (especially access to laptops and reliable internet) remains a challenge^28^, it is evident that technology is pervasive in education today. In fact, it is a significant mediating factor in what, how, where, and when students learn^26^. Access to technology has increased significantly in the past years, especially after the COVID pandemic. Over 94% of children own or have access to touchscreen tablet devices in the UK and USA, and children in low- and middle-income countries in Majority World contexts, such as South Africa, are more likely to have access to a tablet device, compared to a laptop or television^3^.

The role of technology in education has never been more significant than during and post the COVID-19 pandemic. Humans develop technology; hence, technology’s limitations, usability, flaws, and fallacies are linked to human actions. Digital pedagogy through EdTech products and processes can meet diverse student needs. However, choosing suitable technology hinges on contextual factors, like economic, political, cultural, and social conditions. These factors shape EdTech’s policies, implementation, and potential to reduce or exacerbate educational inequalities.

The success of EdTech tools in special needs education suggests their effectiveness in implementing personalized education^39–43^. Research in the learning sciences has shown that every learner learns differently, and significant individual differences in structural and functional brain networks support learning in various domains^34^. EdTech allows students to learn independently using techniques that work best for them.

However, the effectiveness of educational technology hinges on understanding and nurturing the reciprocal relationships between technology and the educational setting. This interconnectedness is vital, as it affects everything from curriculum development to teaching methodologies and student engagement. Technological advancements have given rise to hybrid learning spaces and the metaverse, blurring the boundaries between physical and virtual environments^44,45^. As the world progresses towards artificial intelligence (AI) and mixed reality, educators and learners must develop the necessary competencies to navigate these hybrid learning spaces effectively. However, these spaces also present significant opportunities for innovative curriculum development, teaching methodologies, and assessment practices. They facilitate active and collaborative learning, cognitive apprenticeship, guided exploration, participation in valued knowledge practices, and cultivation of learner autonomy^46–48^. Furthermore, when augmented and virtual reality are integrated with physical spaces, they can create transformative and interconnected learning experiences grounded in real-world contexts^49,50^.

On the other hand, a lack of rigorous and ethical EdTech implementation can lead to algorithm bias, a greater digital divide, misinformation, privacy concerns, and increased teacher apprehension and stress^51^. Education is inherently social, and teacher-student interaction is vital for success. Implementing technology without adequate teacher training and support can negatively affect teacher well-being. Extensive research shows that teacher mental health impacts student outcomes and classroom environments^52–56^. The ISEE Assessment recommends prioritizing teacher well-being, enhancing their social-emotional skills and information literacy, and investing in training to promote collective learning as technology’s role in education expands.

Thus, if technology is to help education solve wicked problems, ethical care and critical thinking should supplement the growing creativity, excitement, and innovations in the field. This approach underscores the necessity of viewing educational technology not just as tools within a classroom but as integral components of a broader educational ecosystem that is dynamic, interconnected and open to external influence.

**Fig. 5:**
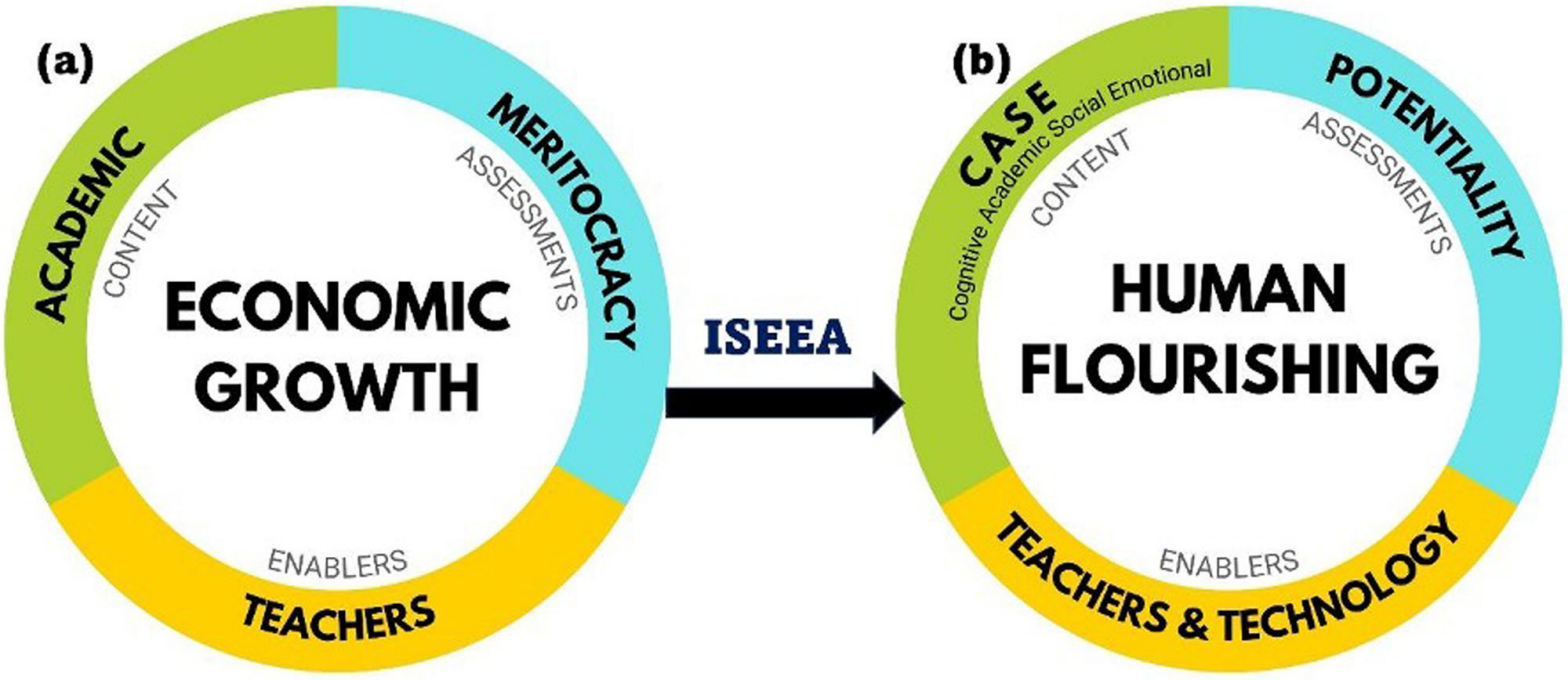
A transformative model of education that shifts its purpose from economic growth to human flourishing. ISEE Assessment’s proposed transformative approach to repurposing education from economic growth **(a)** to **(b)** human flourishing.Part **a** of the figure illustrates the goal of education as economic growth in the global context with a disproportionate emphasis on academic content and meritocracy-driven assessments with teachers as the enablers of building academic knowledge. Part **b** of the figure illustrates ISEE Assessments recommendation of shifting education’s focus to human flourishing using an integrative CASE approach where the development and growth of individual potential is the focus of assessments and wherein teachers and technology act as enablers of human flourishing.

The ISEE Assessment advocates transforming the current education model based on economic growth (Fig. 5(a)), into one centred on human flourishing (Fig. 5(b)) that designs learning, content and assessments through an integrative CASE approach, namely building Cognitive, Academic, Social Emotional skills, repurposing assessments to evaluate potentiality, and envisioning teachers as technology-enabled facilitators who enable every learner to achieve their full potential. The report emphasizes that the brain’s malleability via neuroplasticity^57^ presents a powerful tool for nurturing well-being and peaceful mindsets, fostering these qualities through training and practice, as evidenced by research^54,58–61^.

## Discussion

The ISEE Assessment was the first of its kind in that its primary purpose was to assess education’s impact on developing human potential, fostering individual flourishing according to the Universal Declaration of Human Rights, and promoting peaceful, sustainable societies as outlined in SDG 4.7. The ISEEA possesses distinct features that set it apart from other similar policy initiatives and the discussion below focuses its findings on the context of education policy. Firstly, its definitions of flourishing, learning, teaching and assessment are not only scientifically informed but also deeply rooted in philosophy. and draws on insights from a diverse range of disciplines, that include philosophy, economics, social sciences and neuroscience, to provide a multifaceted understanding of educational flourishing and how it can be promoted. By integrating these various perspectives, the assessment underscores the critical importance of embracing a wide-ranging approach for guiding education in general and global educational policymaking in particular. The third and most compelling aspect is the connection it makes between flourishing, education and research. On the one hand, the ISEE Assessment stresses that rigorous, replicable research should guide education policy, avoiding cherry-picked data and considering local contexts, but it also maintains that education policies should be transparent and evidence-based to create effective pathways and interventions.

This call is part of a broader movement already underway that seeks to reorient education towards well-being and human flourishing^61^, while taking into account the significance of emotions and culture for learning. The work of numerous research initiatives and organizations, such as CASEL^62^, the Science of Learning and Development Alliance, the Positive Education Schools Association^63^, Harvard’s Center on the Developing Child^64^, UQ Learning Lab^65^, and the International Mind, Brain, and Education Society^66^, have already been instrumental in this area. The ISEEA stresses that rigorous, replicable research should guide education policy, avoiding cherry-picked data and considering local contexts. It also maintains that education policies should be transparent and evidence-based to create effective pathways and interventions. Within this broad framework, the ISEE Assessment provides numerous insights and recommendations, which will not be repeated here as they are presented in the Assessment and the Summaty for Decision Makers that followed. Instead, in the remainder of the article, the focus shifts to unique features of the ISEE Assessment not explicitly discussed in the main report. These additional aspects extend beyond the assessment’s specific recommendations into broader educational frameworks and practices, with the aim of enriching our understanding and offering guidance for shaping future educational policy to support human flourishing better.

The first distinct feature of the ISEE Assessment is its definition of flourishing, which is anchored in both scientific evidence and philosophical throries. While concepts of flourishing and well-being are becoming more common in educational policy reports—many of which aim to be grounded in science—few purposefully and explicitly incorporate normative perspectives^67^. By defining flourishing through a philosophically informed lens, the ISEE Assessment addresses dimensions that traditional conceptions of science may overlook, an approach essential to capturing the full scope of human flourishing. This philosophical grounding is not only crucial for understanding flourishing but is also important for education as a whole, as education is fundamentally a normative endeavour. Retaining this normative dimension enriches the educational process and supports a broader vision of human development.

Secondly, the ISEE Assessment distinguishes itself through the breadth and depth of the issues it addresses. It spans the micro, meso, and macro levels of education (Fig. 2) and draws on insights from a diverse range of disciplines, including philosophy, economics, and neuroscience, to provide a multifaceted understanding of educational flourishing and how it can be promoted. By integrating these varied perspectives, the assessment takes a holistic approach, acknowledging the complex interplay of factors that shape educational outcomes. This wide-ranging approach is essential for guiding education broadly and global educational policymaking, in particular, ensuring that policies consider the full spectrum of influences on learning and well-being. It also emphasizes the need to prioritize diverse research, bridging the Global South and the Global North and consider unique sociocultural contexts.

The third and most compelling unique feature of the ISSE Assessment is the nuanced connection it makes between flourishing, education and research. On the one hand, the ISEE Assessment stresses that rigorous, replicable research should guide education policy and cautions against cherry-picked data that suits and considers only local contexts. It also maintains that education policies should be transparent and evidence-based to create effective pathways and interventions. The assessment also discusses the question of what constitutes science and evidence in education at length. ISEE Assessment follows the definition of science as put forward by The Science Council, which is “the pursuit and application of knowledge and understanding of the natural and social world following a systematic methodology based on evidence^67^.’ ISEE Assessment conceptualises ‘relative evidence’ as ‘the result of thorough comparisons of extant interventions, under the assumption that combined results coming from meta-analyses or systematic reviews are much more informative than single – albeit excellent – studies, when necessary, precautions are taken^68,69^.’ When considering the evidence for policy decisions, it is important that reviewed evidence is critically considered to deduce if they are probative, scientific or pseudo-scientific and non-scientific beliefs^69^.

At the same time, the assessment acknowledges that education is a complex system intricately linked with other systems such as economics, cultural norms, law, and governance. It also portrays education as a dynamic entity heavily influenced by local contexts. As demonstrated in this article, through the three recommendations examined, the ISEE assessment advocates for numerous principles derived from this outlook. It promotes a holistic understanding, emphasising interrelations, highlighting the importance of interconnections and mutual influences, and stressing the significance of both the environment and context. It thereby broadens the understanding of what constitutes scientific approaches in education. This contributes to a more comprehensive understanding of education and human flourishing, extending beyond traditional global policy approaches. By advocating for a collective educational goal based on such a broad view of science and evidence, the ISEE Assessment suggests that we can best serve both humanity and the planet, ultimately fostering peaceful and sustainable societies.

## Methods

The conceptual framework that forms the cornerstone of the ISEE Assessment is built on Aristotle’s concept of eudaimonia, loosely translated as “flourishing.” This concept was selected following an exhaustive review of the literature, which established its significance across various fields, from philosophy and education to economics. From the literature review and subsequent discussions among the report’s authors, a working understanding of “flourishing” emerged that is suitable for guiding educational aims. This understanding was then examined and compared to the concept of human capital, traditionally considered the primary educational goal for securing economic growth and well-being. The adopted definition of flourishing rests on the idea that, since humans are social-emotional beings, education must ensure the holistic development of the human brain, nurturing both human relationships and academic growth for overall well-being. Additionally, this definition aligns with Article 26 of the Declaration of Human Rights, which states that education should enable individuals to develop their full potential. These three factors are captured in the description of human flourishing (Fig. 3) and form the heart of education systems. Consequently, the ISEE Assessment report adopts a unique approach by focusing on human flourishing^22–24^.

As explained in the position paper^20^, the research for the ISEE Assessment was conducted in four working groups, each focusing on a specific aspect.

Working Group 1 assessed education’s alignment with flourishing, established assessment benchmarks, defined key terms, and examined various dimensions of integrating flourishing into education.

Working Group 2 assessed the impact of macro-level factors (economic, social, cultural, political) on education institutionally and individually (e.g., learning, teaching, assessment).

Working Group 3 examined the micro-level factors (e.g., socioeconomic status, culture, biology) impacting individual learning processes and shaping unique learning traits.

Working Group 4 sought to establish benchmarks on what constitutes data and evidence within the education sector. This was driven by the need for more consensus among ISSEA experts from various disciplines.

The transdisciplinary research review and consensual discussions among working groups 1, 2 and 3 led to some fundamental descriptors against which the assessment was carried out (Fig. 3).

## Data Availability

There are no custom codes associated with this paper.
